# The Many Faces of Intestinal Tumors in Adults, Including the Primary Role of CT Imaging in Emergencies and the Important Role of Cross-Sectional Imaging: A Pictorial Review

**DOI:** 10.3390/healthcare13091071

**Published:** 2025-05-06

**Authors:** Barbara Brogna, Francesca Maccioni, Dolores Sgambato, Fabiana Capuano, Lorenzo Iovine, Salvatore Guarino, Lorenzo Di Libero, Alfonso Amendola, Lorenzo Faggioni, Dania Cioni

**Affiliations:** 1 Unit Interventional and Emergency Radiology, St. Giuseppe Moscati Hospital, Center of National Excellence and High Speciality, 83100 Avellino, Italy; 2 Department of Radiological, Oncological and Pathological Sciences, Umberto I Hospital, Sapienza University of Rome, Viale Regina Elena 324, 00161 Rome, Italy; francesca.maccioni@uniroma1.it; 3 Division of Gastroenterology, St. Giuseppe Moscati Hospital, Center of National Excellence and High Specialty, 83100 Avellino, Italy; 4 Department of Surgery, Responsible Research Hospital, Largo A. Gemelli, 86100 Campobasso, Italy; 5 Department of Radiology, Monaldi Hospital, AORN dei Colli, Str. Vicinale Reggente 66/82, 80131 Naples, Italy; 6 Department of General and Specialist Surgery, St. Giuseppe Moscati Hospital, Center of National Excellence and High Specialty, 83100 Avellino, Italy; 7 Oncological and General Surgery Unit, St. Giuseppe Moscati Hospital, Center of National Excellence and High Specialty, 83100 Avellino, Italy; 8 Academic Radiology, Department of Translational Research, University of Pisa, Via Roma, 67, 56126 Pisa, Italy; 9 Academic Radiology, Department of Surgical, Medical, Molecular Pathology and Emergency Medicine, University of Pisa, Via Roma, 67, 56126 Pisa, Italy

**Keywords:** small bowel tumors, emergency, CT, cross-sectional imaging, complications, artificial intelligence

## Abstract

**Background/Objectives:** Small bowel tumors (SBTs) encompass a diverse range of tumor types, with benign tumors being the most prevalent. However, the incidence of malignant SBTs is increasing, particularly small bowel adenocarcinoma; this poses a diagnostic challenge for clinicians and radiologists due to the varied and nonspecific clinical and radiological presentations associated with SBTs. In fact, SBTs can present differently in emergencies, often mimicking inflammatory diseases or manifesting as complications such as intussusception, small bowel obstruction (SBO), intestinal ischemia, perforation, gastrointestinal bleeding, or metastatic disease. These tumors can remain asymptomatic for extended periods. **Methods:** We present a pictorial review on the role of imaging in evaluating SBTs, focusing on the emergency setting where diagnosis can be incidental. We also include some representative cases that may be useful for radiologists and residents in clinical practice. **Results**: Despite these challenges, contrast-enhanced computed tomography (CECT) is usually the best modality to use in emergencies for evaluating SBTs, and in some cases, a diagnosis can be made incidentally. However, when possible, multimodal imaging through cross-sectional imaging remains crucial for the non-invasive diagnosis of SBTs in stable patients, as endoscopic procedures may also be impractical. A complementary CT study with distension using negative oral contrast media, such as water, polyethylene glycol, or mannitol solutions, can improve the characterization of SBTs and rule out multiple SBT locations, particularly in small bowel neuroendocrine tumor (NET) and gastrointestinal tumor (GIST) localization. Positive water-soluble iodine-based oral contrast, such as Gastrografin (GGF), can be used to evaluate and monitor the intestinal lumen during the nonsurgical management of small bowel obstruction (SBO) or in suspected cases of small bowel perforations or the presence of fistulas. Magnetic resonance enterography (MRE) can aid in improving the characterization of SBTs through a multiplanar and multisequence study. Positron emission tomography combined with CT is generally an essential modality in evaluating metastatic disease and staging and assessing tumor prognosis, but it has limitations for indolent lymphoma and small NETs. **Conclusions:** Therefore, the integration of multiple imaging modalities can improve patient management and provide a preoperative risk assessment with prognostic and predictive indicators. In the future, radiomics could potentially serve as a “virtual biopsy” for SBTs, allowing for better diagnosis and more personalized management in precision medicine.

## 1. Introduction

Primary small bowel tumors (SBTs) encompass a wide variety of tumors, with benign tumors being more prevalent. However, in recent years, there has been an overall increase in reported malignant SBTs. This increased incidence is linked to unhealthy lifestyle habits such as alcohol consumption and smoking [1,2,3], metabolic disorders, celiac disease (CD), and chronic inflammatory bowel disease (IBD), such as Crohn’s disease (CHD) [1,2,3,4,5]. Numerous studies provide evidence that chronic inflammation increases the risk of cancer, promotes tumor progression, and supports metastatic spread through multifaceted mechanisms [4,5,6,7,8,9,10].

Inflammatory mediators such as cytokines (TNF-α, IL-1β, IL-6, IL-10), reactive oxygen species (ROS), and reactive nitrogen species (RNS) can induce epigenetic alterations in premalignant lesions and silence tumor suppressor genes [6,9,10,11,12]. In chronic inflammation, IL-6 increases the survival of neutrophils by reducing apoptosis, and IL-6 also promotes cytokine secretion through Th1, Th2, or Th17 cells [4,7,10]. IL-1β and IL-6 are important cytokines for both intestinal inflammation and colorectal cancer [9,12]. IL-10 has an important role in controlling immunological gut homeostasis [9,12]. Moreover, genetic variations of IL-10 are associated with the early onset of IBD [9,10,11,12]. Pavel et al. [12] provided immunohistochemical evidence linking chronic inflammation in IBD to dysplasia and cytokine-driven disease progression.

Prolonged inflammation also disrupts normal tissue repair processes, weakening the epithelial barrier and altering microbial components through the production of toxins and promotion of tumorigenesis [4,5,7].

Other risk factors associated with an increased incidence of small bowel tumors include some hereditary conditions such as familial adenomatous polyposis (FAP), Lynch syndrome (LS), and Peutz–Jeghers syndrome (PJS) [5,13,14,15]. Due to their rarity, primary SBTs continue to present challenges in healthcare practice as clinical presentations may be nonspecific and early diagnosis remains difficult, especially for malignant tumors [5,14,15,16,17,18]. Furthermore, many patients present with vague clinical symptoms such as mild abdominal pain over a long period, weight loss, or unexplained iron anemia, and can present to the emergency clinic with complications or advanced disease and poor prognosis [5,14,15,16,17,18]. It is still very difficult to comprehensively study the loops of the small intestine in terms of their extension and angulation, even with endoscopic techniques, which are limited to evaluating the mucosa [19,20]. In addition, capsule endoscopy cannot be performed in the presence of occlusive or sub-occlusive symptoms, while double-balloon endoscopy can be compromised in the presence of adhesions or stenosis [20,21].

Imaging plays a crucial role in supporting the diagnosis of SBTs. This is not only due to its non-invasiveness but also because, in emergency settings, only imaging can support the diagnosis and guide therapeutic management, including surgical intervention [14,15,16,17,18,22]. Surgery is usually curative in localized diseases. Therefore, earlier detection, even if made incidentally, can improve patient management and avoid complications [14,17,18,19]. A correct diagnosis can be made based on the location of the tumor and its pathogenesis, which leads to specific radiological patterns [23]. Malignant lesions, such as adenocarcinomas and carcinoids, make up the majority of small bowel cancers [23,24]. Adenoma, adenocarcinoma, and NETs typically arise from the epithelial layer [23]. Small bowel NETs usually develop from enterochromaffin cells; these are endocrine cells located in the small bowel epithelium, which secrete the majority of the body’s serotonin [25,26,27,28]. Carcinoids are the most common small bowel NETs [25,26,27,28]. Based on its location, small bowel adenocarcinoma occurs more frequently in the duodenum and jejunum. However, adenocarcinoma can also occur as a rare complication of long-standing Crohn’s disease, in which case it is more likely to occur in the ileum [23,24,29]. Adenocarcinoma typically presents as a short-segment, irregular wall thickening of the small bowel [23,24,30]. Small bowel carcinoids are more frequent in the ileum and can also have multiple localizations [25,26,27,28]. They can present as polypoid or plaque-like masses with mesenteric infiltration, causing a desmoplastic reaction and small bowel angulation [25,26,27,28]. The desmoplastic reaction in small bowel carcinoids can also be associated with calcification [25,26,27,28].

Other tumors, including lymphoma, lipoma, hemangioma, and inflammatory fibroid polyps, arise in the submucosal layer [23,24,31]. Intestinal lymphoma is usually more frequent in the ileum and usually involves long or multiple bowel segments [23,24,31]. However, in patients with celiac disease, there is an increased incidence of T-cell lymphoma in the small bowel, especially in the jejunum [23]. Small bowel lymphoma may present as a polypoid mass, multiple nodules, an extraluminal mass, or extensive involvement of the bowel wall, causing mural or mucosal thickening that leads to strictures or the aneurysmal dilatation of the bowel lumen. Furthermore, small bowel lymphoma is usually associated with enlarged mesenteric lymph nodes [23,24,31].

Gastrointestinal stromal tumors (GISTs) usually develop from the interstitial cells of Cajal in the muscularis propria, while metastasis can involve various layers of the small bowel [23].

GISTs are usually more common in the jejunum than in the ileum and can also have a multifocal presentation, especially in neurofibromatosis [23,24,32,33].

GISTs can exhibit an intramural, pedunculated, endoenteric, exoenteric, or mixed growth pattern [23,24,33]. Intratumoral calcifications can also be found in GISTs [23,33]. Small bowel inflammatory fibroid polyps can present as focal parietal thickness [23].

Metastasis can involve the various layers of the small bowel according to the spreading mechanism [23,24]. They can present as bulky mesenteric masses, which mimic lymphoma, or rarely as focal bowel wall thickening, similar to adenocarcinoma [23,24,34].

## 2. Updates on the Role of Imaging in the Evaluation of Small Bowel Tumors

### 2.1. Small Bowel Tumors: The Role of Imaging in Emergencies and the Importance of Cross-Sectional Imaging Studies

**Figure 1:**
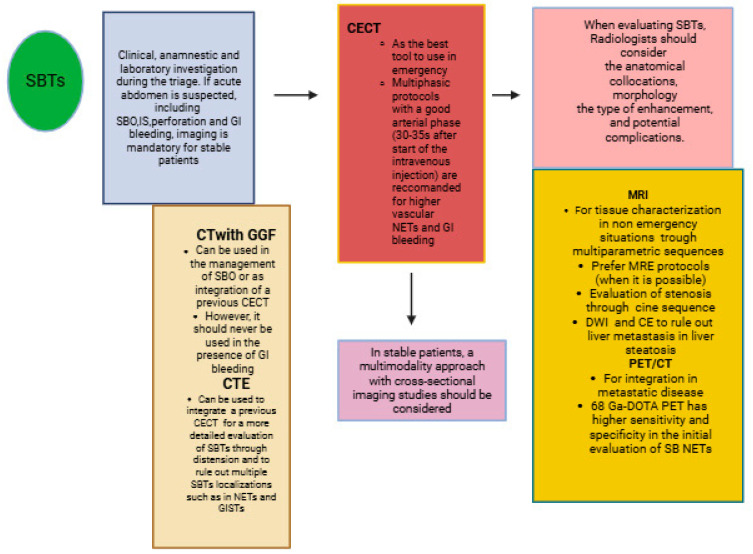
The summary of the role of each imaging modality in the diagnosis of SBTs.

SBTs can present with a variety of symptoms in the emergency setting, mimicking inflammatory diseases or manifesting as complications such as acute abdomen with obstructive symptoms, gastrointestinal bleeding, ischemia, and perforation [18,19,31]. Generally, careful clinical and laboratory investigations during triage, along with imaging studies, are mandatory to evaluate SBTs, as diagnoses can sometimes be made incidentally. Nevertheless, cross-sectional imaging studies are very helpful for tumor characterization in stable patients [16,34,35] (Figure 1). The integration of information from different imaging modalities can improve patient management and provide a preoperative risk assessment [16,34,35]. It is important to provide surgeons with all of the necessary information, such as SBT localization, contrast enhancement, and potential complications, to ensure successful surgical resection (R0) of the primary tumor and locoregional lymph nodes [14,15,16,17,18,19]. This represents the only potentially curative treatment for localized disease. In the metastatic setting, primary tumor resection is generally not recommended unless there is acute bowel obstruction, perforation, or uncontrolled bleeding [14,15,16,17,18,19]. Therefore, radiologists should be aware of the various presentations of SBTs in emergency settings and the different imaging modalities available based on the clinical situation to determine the most appropriate surgical or therapeutic management.

Abdominal contrast-enhanced computed tomography (CECT) is usually the preferred diagnostic tool in emergency settings due to its accessibility and speed, making it the modality of choice for patients in poor clinical condition [23,24]. Multidetector CT (MDCT) enables the exploration of the gastrointestinal (GI) tract, acquiring a volume data set of the abdomen and, through multiplanar reconstruction (MPR), provides a three-dimensional and panoramic evaluation of the small bowel loops [23,24,36]. By using neutral oral contrast media, such as water polyethylene glycol or mannitol solutions, CT can enhance the contrast between the lumen and the small bowel wall, aiding in the assessment of mucosal thickening and wall stratification/enhancement patterns [37,38,39]. Water alone is often insufficient for proper distension due to rapid reabsorption [37,38].

Through small bowel lumen distension, CT enterography (CTE) has been shown to improve the detection of small SBTs and the localization of multiple tumors [37,38,39]. Both small GISTs and NETs frequently exhibit a characteristic arterial hypervascular enhancement pattern on CECT [37,38,39]. However, CTE is less sensitive than endoscopic studies for the detection of small mucosal lesions [37]. Alternatively, positive oral contrast agents, such as barium-based oral contrast or water-soluble iodine-based oral contrast, such as Gastrografin (GGF), can be used to evaluate and monitor the intestinal lumen during the nonsurgical management of small bowel occlusion (SB) [40,41]. While barium-based oral contrast is generally contraindicated in cases of suspected bowel perforation due to the risk of barium peritonitis, GGF and iodine-based oral contrast can be beneficial in suspected cases of small bowel perforations or in the presence of fistulas [40]. However, positive oral contrast should never be used in cases of suspected GI bleeding as it can mask endoluminal bleeding [40,41]. Nevertheless, advancements in CT technology, such as dual-energy computed tomography (DECT) techniques, have further improved the detection and characterization of SBTs, using quantitative parameters such as iodine concentration [42,43,44,45]. On the other hand, DECT also improves the detection of GI bleeding caused by tumors with low-energy monochromatic images that enhance the conspicuity of areas of active bleeding [44,45]. The use of low-energy images or iodine maps may also improve hypervascular lesion detection in NETs and GISTs and can also be used to better delineate bowel masses or serosal bowel lesions due to their ability to accentuate areas of increased uptake of iodinated contrast material [42,43,44,45]. The use of abdominal X-rays in the study of SBTs is usually limited to emergency cases of SBTs presenting with SBO or perforation [24]. Intestinal ultrasound (IUS) typically allows for the evaluation of intestinal motility, and its role is increasing in the initial evaluation of patients with CHD, especially in the pediatric population. However, its role in the detection of SBTs is limited by several factors, such as the physician’s experience, the presence of intestinal air, and a higher body mass index [46,47,48,49]. Small bowel motility issues can be addressed using MRI enterography (MRE), which provides a complete evaluation of the entire intestinal tract. Through multiparametric and multisequence evaluation, MRE can distinguish between the benign and malignant nature of SBTs [24,25,50,51,52,53,54,55,56,57]. MRE typically requires intestinal distension with oral biphasic contrast agents, such as mannitol, PEG, sorbitol, or lactulose, with an optimal volume of 1000–1500 mL that can be ingested over 45–60 min before the examination [54,55,56,57]. This way, through distension, MRE improves the detection of small intestinal tumors. When possible, it should be performed in the prone position to reduce artifacts from respiratory motions and peristalsis [54,55,56,57]. MRE sequences have evolved over time to include rapid imaging acquisition, high resolution, and a wide field of view, which are three crucial requirements for the satisfactory evaluation of the small bowel. Breath holding and fast imaging are essential for bowel evaluation, along with good to high spatial resolution and a wide field of view to assess the entire small bowel [54,55,56,57]. The MRI sequences typically included in MRE protocols, based on European and American guidelines, are fast sequences, such as axial and coronal single-shot fast spin echo (SS-FSE/HASTE) with and without fat suppression; axial and coronal steady-state free procession (FIESTA, True-FISP) without fat suppression; and axial and coronal pre- and post-3DT1-weighted gradient-echo sequences with fat suppression [54,55,56,57]. The use of spasmolytic agents such as hyoscine butylbromide is usually recommended in fractional or single doses before motion-sensitive sequences, such as T2-weighted and post-contrast T1 sequences, to improve small bowel distension and reduce peristalsis motion artifacts [54,55,56,57]. The application of DWI sequences can help determine the grade of malignant SBTs and provide useful information about treatment response [54,55,56,57,58,59]. Intestinal lymphomas are typically associated with higher DWI restriction and lower values on ADC maps [58,59]. If a patient’s compliance is not acceptable in the prone position, the supine position may be useful, and if MRE cannot be performed, MRI without distensions may be helpful in characterizing SBTs and in patients allergic to CT contrast medium [54]. Additionally, MRE also plays an important role in intestinal polyp surveillance, particularly in patients with PJS [54,60]. It has been reported that a combination of supine and prone positions is significantly more accurate than the supine position alone for detecting polyps smaller than 15 mm [54,60].

Positron emission tomography (PET) combined with CT has generally become an essential modality in the evaluation of metastatic disease, staging, and the assessment of tumor prognosis [61,62,63,64]. However, some problems can occur in small bowel evaluation as the majority of commonly used radiotracers show physiologic small bowel avidity, which is due to either hepatobiliary excretion or active secretion into the lumen [61]. Additionally, small bowel movement can cause the misregistration of PET and CT images, making it difficult to localize 2-deoxy-2-^18^F-D-glucose (FDG) activity [61,62]. Indolent lymphoma and small bowel NETs can be easily missed on PET/CT as they may show a mild uptake of FDG [61,62]. Small bowel adenocarcinomas usually show an increased uptake of FDG, but, in some cases, small bowel adenomas and adenocarcinomas may be difficult to distinguish on PET/CT [61]. Small bowel high-grade lymphomas are usually associated with higher FDG avidity, as well as poorly differentiated small bowel NETs showing higher FDG avidity on PET [61]. In contrast, small bowel NETs can be easily missed on PET FDG, especially when they have a low proliferation index of less than 2% [61]. On the other hand, for small bowel NETs, 68-Gallium (68Ga)-DOTA peptide positron emission tomography (PET/CT) has higher sensitivity and specificity for tumor localization, staging, and receptor status assessment [61,62,63,64,65]. 68-Gallium DOTA PET/CT is a type of functional imaging that uses different radioisotope-labeled somatostatin analog peptides such as DOTATATE, DOTATOC, and DOTANOC, which bind to the somatostatin receptor found in NETs. 68-Gallium DOTATATE PET/CT can be useful in patients who have carcinoid-like symptoms or are biochemically positive for NETs but with negative anatomic imaging and endoscopy studies [66]. In a study by Bonomi et al. [66], the combined use of CTE and 68 Ga DOTATATE PET/CT was found to be useful in identifying low-risk surgical candidates with small bowel NETs. Malignant small bowel GISTs are usually hypermetabolic on PET/CT [61]. Therefore, the multimodal approach in stable patients can be used to gather information from various imaging modalities. This information can then be integrated, like pieces of a puzzle, to improve tumor characterization and align predictive and prognostic indicators.

Table 1 summarizes the main clinical indications, including the protocols, advantages, and limitations of each imaging modality.

### 2.2. Small Bowel Tumors: The Emerging Role of Artificial Intelligence and Radiomics

Technological advancements, coupled with the increasing use and improvement of artificial intelligence (AI) applications, will deeply impact healthcare management in the near future [67,68,69]. This will allow for better diagnosis and more personalized management in precision medicine. Several studies have shown that AI in cancer imaging can be used to improve tumor detection, characterization, and the monitoring of tumor response through deep learning (DL) models [67,68]. Moreover, AI can be applied to radiomics features, providing rapid and non-invasive biomarkers for cancer diagnosis and prognosis [67,69]. There have been several advancements in the application of AI for the detection of SBTs through endoscopy [70,71,72]. AI can improve the evaluation of SBTs through capsule endoscopy, reducing the interference of intestinal content [70]. However, due to the heterogeneity and rarity of SBTs, there are still few imaging studies on the application of AI in SBT evaluations. Nevertheless, AI has great potential in GI oncology. Numerous AI-assisted models have emerged in research on gastric, esophageal, and colorectal cancers (CRCs) [73,74,75,76]. There is also growing interest in radiomics applications for SBTs. Recent studies have focused on advancements in radiomics applications for differential GIST diagnosis from other GI neoplasms, risk stratification, and prognosis prediction after surgery [77,78,79,80]. Radiomics has immense potential to improve knowledge in SBT biology and to identify imaging biomarkers that can contribute to their detection, optimal therapeutic strategy, prognosis, prediction of response, and surveillance [66,67,68,69,70,71,72,73,74,75,76,77,78,79,80]. However, the application of AI-based methods in cancer imaging has not been standardized to date, and there are differences in study methodology and in the performance of radiomic models [67,69]. In the future, radiomics could potentially serve as a “virtual biopsy” for SBTs in clinical practice.

### 2.3. Small Bowel Tumor Presentation in Emergencies with Intestinal Intussusception

Intussusception (IS) is defined as the invagination of a bowel segment into an immediately adjacent bowel segment. It is usually caused by an alteration in peristalsis, which may be idiopathic, anatomical, benign, or malignant. It occurs more frequently in the small bowel than the large bowel and is more common in children. However, while the cause of IS in children is usually idiopathic and benign (infectious etiology, anatomic intestinal abnormalities), in adults, IS can frequently be caused by intestinal tumors and can present as an emergency condition manifesting with acute abdomen and SBO [81,82,83,84,85,86].

**Figure 2:**
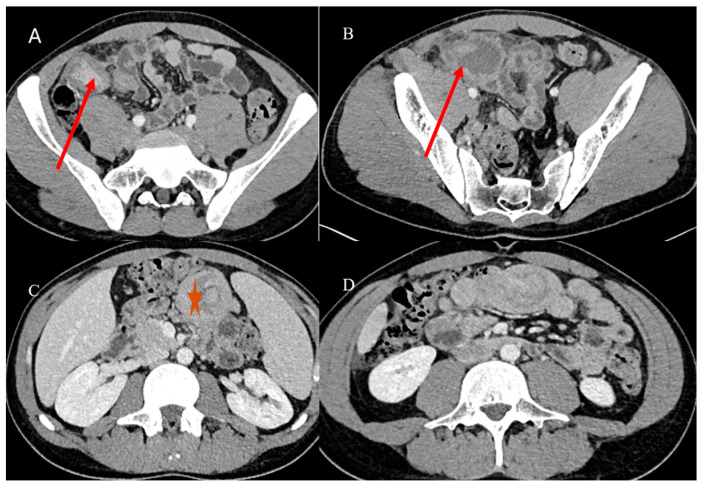
A 28-year-old man came to the emergency room with acute abdominal pain. Images (**A**,**B**) show the abdominal CECT with abscessed appendicitis (red arrow). However, incidentally, on the CECT, a proximal jejunal IS was also found, as represented in images (**C**) (orange star) and (**D**). The jejunal invagination was caused by a Peutz-Jeghers (PJ) polyp.

Adult IS represents 1% to 5% of all causes of SBO [81]. Abdominal CT is the best tool for emergencies and can be useful in diagnosing the underlying causes of IS, with a reported accuracy of 58–100% [81,82,83,84,85]. With CT, it is possible to identify the leading point of IS, which is usually associated with benign or malignant lesions, as well as the location and extent of the IS [81,82,83,84,85]. However, sometimes the diagnosis of IS can be made incidentally on CT in patients presenting for other reasons or other emergencies, as in acute appendicitis [87,88] (Figure 2).

**Figure 3:**
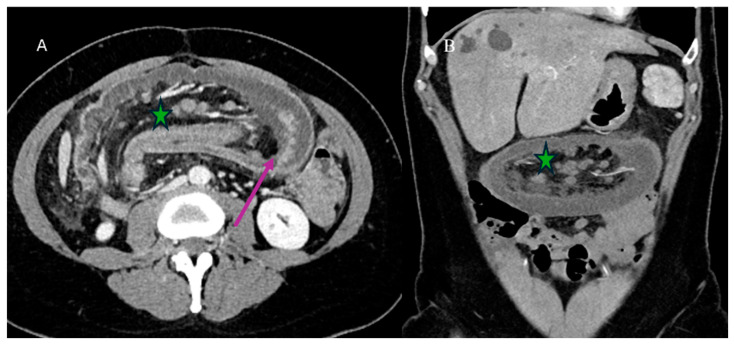
An abdominal CECT was performed on a 58-year-old woman who came to the emergency room with acute abdomen. The scan revealed an ileocecal intussusception caused by an ileal adenocarcinoma. Image (**A**) shows the ileocecal IS with irregular neoplastic thickness (violet arrow) and lymphadenopathy (green star) on the axial plane. Image (**B**) depicts the ileocecal intussusception with lymphadenopathy (green star) on the coronal plane.

Nevertheless, the typical features of IS with a leading point include an abnormal target-like or sausage-shaped mass with a cross-sectional diameter greater than that of the normal bowel. This may be associated with proximal bowel obstruction [81,82,83,84,85]. On CECT, it is possible to visualize the type of enhancement of the outer intussuscipiens and the central intussusceptum, creating a bowel-within-bowel appearance with the mesenteric hypodense fat in the intermediate layer [81,82,83,84,85]. The findings associated with malignancy include the presence of an irregular mass in the central intussusceptum and associated lymphadenopathy [81,82,83,84,85] (Figure 3).

Additionally, CECT can visualize vascular perfusion in terms of venous stasis and the presence of edema, air, and possible complications, such as necrosis, gangrene, and obstruction [81,82,83,84,85].

**Figure 4:**
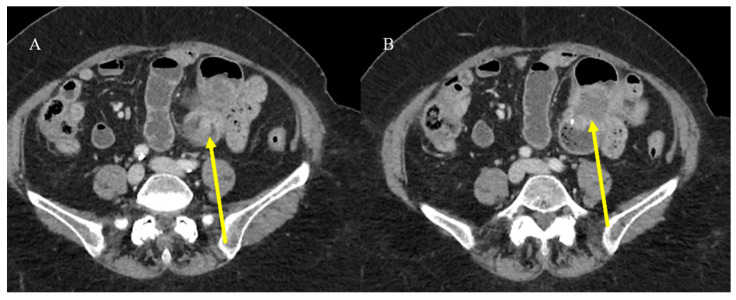
A 72-year-old woman came to the emergency room with abdominal pain with sub-occlusive symptoms. Image (**A**) shows a focal invagination caused by an intestinal polyp (yellow arrow). In image (**B**), the polyp is highly visible as a hypodense formation in the ileal wall (yellow arrow).

Based on location, IS may be classified as enteroenteric, ileocolic, ileocecal, or colocolic. In adults, intussusception with a lead point tends to be persistent or recurrent, but it can also be transient. Benign tumors, particularly intraluminal polypoid lesions, have a greater tendency to cause IS [81,82,83,84,85,86] (Figure 2 and Figure 4).

**Figure 5:**
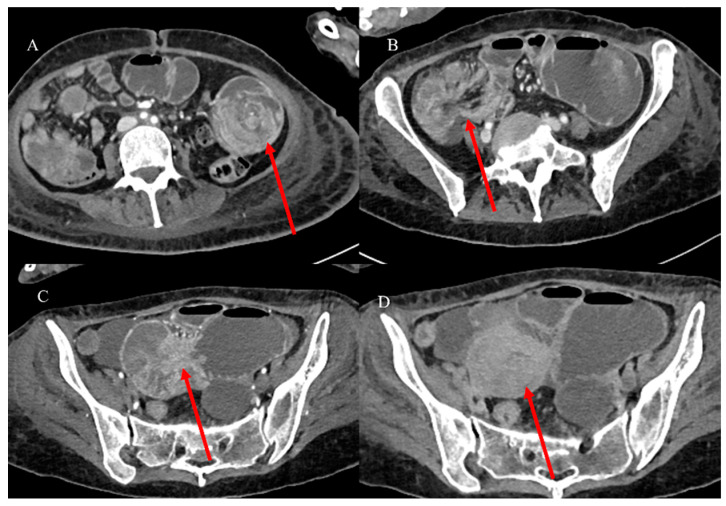
The abdominal CECT of a 53-year-old woman with metastatic melanoma revealed multiple intestinal IS caused by intestinal metastasis. Image (**A**) shows a duodenal proximal metastatic IS (red arrow), while image (**B**) depicts an ileal distal IS. Additionally, images (**C**,**D**) show another ileal IS (red arrow).

Less commonly, malignant tumors may act as lead points, with metastatic disease being the most common cause [23]. Approximately 50% of malignant lesions causing intussusception are metastatic (miliary) melanomas [23] (Figure 5).

**Figure 6:**
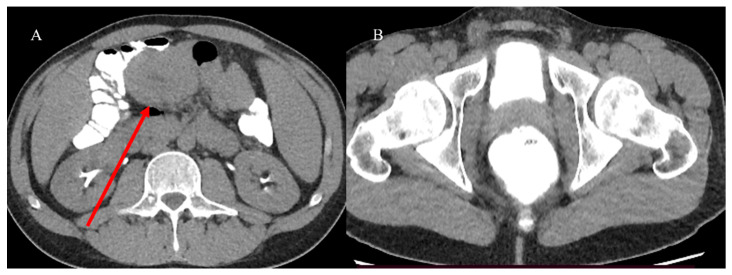
The CT scan shows integration with GGF in the previous case of a 28-year-old man with appendicitis and IS. The invagination was partially resolved (red arrow) in image (**A**), and the GGF progressed in the rectum (**B**).

**Figure 7:**
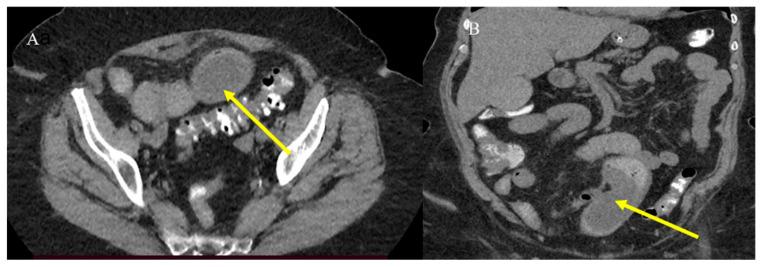
The CT scan shows integration with GGF in the previous case of the 72-year-old woman with sub-occlusive symptoms and an intestinal polyp. The intestinal polyp is highly visible in images (**A**,**B**) after the small bowel lumen opacification by GGF as an ipodense mass (yellow arrow), and the sub-occlusive symptoms were resolved as the GGF progressed in the sigma and rectum.

The malignant intraluminal causes of IS include primary adenocarcinoma, GISTs, carcinoid tumors, neuroendocrine tumors, and lymphomas [81,82,83,84,85,86]. However, visualizing the lead point and the presence of a mass associated with IS can be challenging, especially if there is bowel wall edema due to impaired circulation of the mesenteric vessels [81,82,83,84,85,86]. Therefore, it can be difficult to differentiate a lead mass from inflammation [81,82]. In cases of IS associated with SBO or to distinguish a transient intussusception from one with a lead point, integrating the study with positive oral contrast, such as GGF, may have a therapeutic role and guide surgical management [81] (Figure 6 and Figure 7).

**Figure 8:**
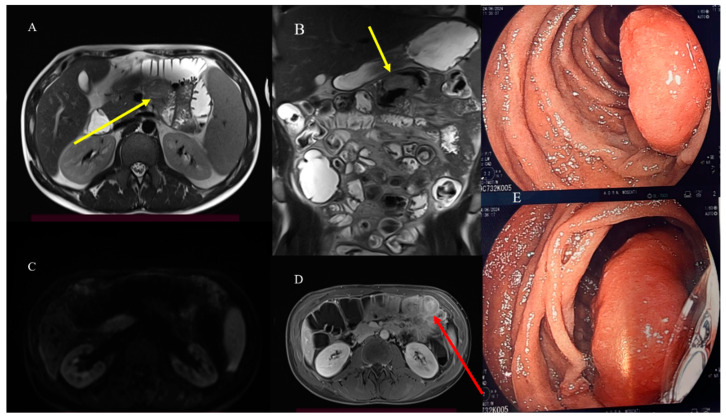
MRI of the 28-year-old man who came to the emergency room with appendicitis and intestinal invagination. Images (**A**,**B**) show the T2 HASTE MRI sequence where a mild hypointense formation of 3 cm was visible in a jejunal loop immediately after the Treitz (yellow arrow). In image (**C**), the hypointense intestinal endophytic formation does not show a diffusion restriction in the DWI sequence. However, a focal invagination is still visible in the T1 vibe Dixon sequence after contrast administration (red arrow) in image (**D**). This formation was compatible with an intestinal polyp and was confirmed in an endoscopy study using gastroscopy, as depicted in image (**E**). Histology confirmed a PJ polyp.

MRE may also be useful in characterizing the underlying causes of IS as benign and malignant tumors in non-emergency situations [81] (Figure 8).

### 2.4. Small Bowel Tumors Causing Small Bowel Occlusion

**Figure 9:**
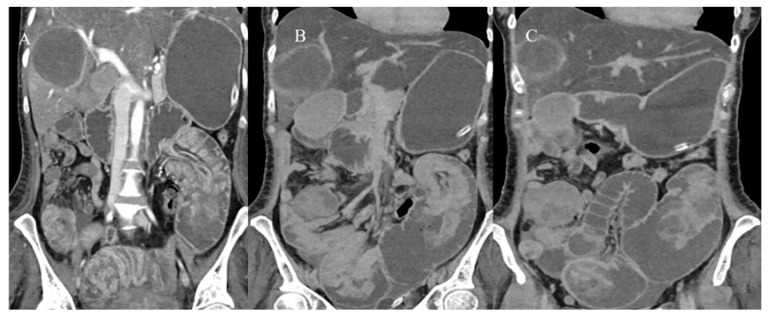
The CECT coronal plane images depict multiple intestinal invaginations with metastatic melanoma and SBO in images (**A**–**C**). Liver metastases were also visible, along with severe steatosis.

SBTs can be a rare cause of SBO in emergency settings. This obstruction can result from intraluminal occlusion due to the tumor’s growth or infiltration through the mucosa, leading to the obstruction of the lumen or the impairment of peristaltic movements [89]. Tumors involving the mesentery and omentum can also cause extramural bowel occlusion by angulating the bowel [89]. The infiltration of the enteric or celiac plexus can severely impair peristalsis, leading to obstruction due to dysmotility [89]. Adenocarcinoma and neuroendocrine tumors are the most common SBTs associated with SBO, as reported in various case studies [89,90,91,92,93]. Small bowel metastases can occasionally present with SBO, which may also be secondary to small bowel IS [94,95,96,97] (Figure 9).

**Figure 10:**
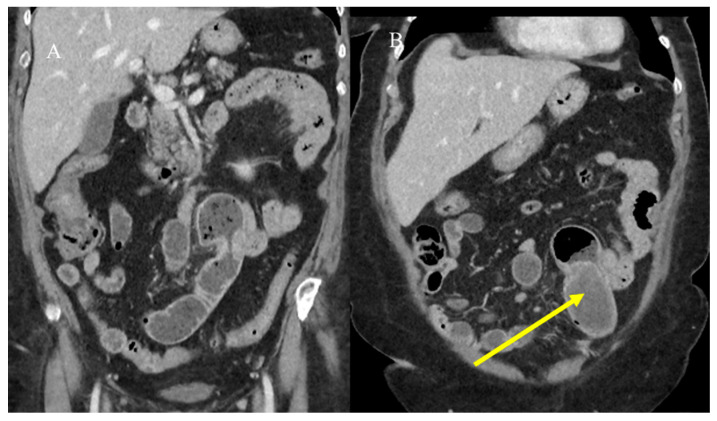
The CECT coronal plane images of the previous case of a 72-year-old woman with an intestinal polyp and SBO. Mild distension of the intestinal loops is visible in image (**A**) with the intestinal polyp highlighted in image (**B**) (yellow arrow).

In addition, benign tumors can present in emergency settings with SBO and can also be secondary to IS [98,99] (Figure 10).

Lymphoma is less likely to cause SBO as it tends to expand the small bowel loops, and the muscle layer can be replaced by the lymphoid tissue [23,24,31]. However, the stenosing form may be encountered in patients with CD [23,24].

Abdominal X-ray is typically the first imaging tool used in cases of SBO [100,101]. However, its overall accuracy in diagnosing SBO is usually low and it cannot provide a clear etiology of the obstruction. In contrast, MDCT is the preferred imaging tool in emergency cases of SBO, with a sensitivity and specificity of 95% for diagnosing high-grade SBO [89,100,101].

**Figure 11:**
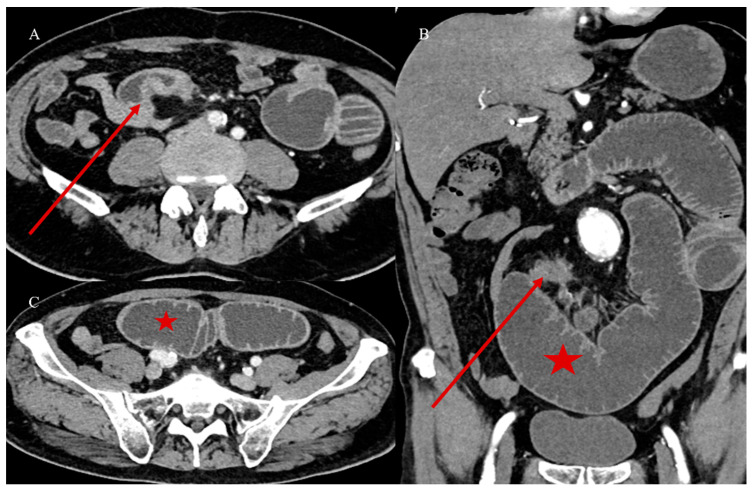
The abdominal CECT of a 72-year-old man who presented in the emergency room with acute abdomen and SBO revealed an irregular stenotic thickness at the level of a distal jejunal loop, as seen in image (**A**). Additionally, image (**B**) displays a coronal plane where irregular tissue in the nearby mesenteric fat (red arrow) is clearly visible, with a high grade of obstruction (red star) as a sign of persistent blockage. This high grade of obstruction is also evident on the axial plane in image (**C**). The imaging findings suggest neoplastic stenosis from adenocarcinoma, with a differential diagnosis of a carcinoid tumor. Histology confirmed intestinal adenocarcinoma with infiltration of the mesenteric tissue.

On CT, it is possible to visualize the neoplastic stenosis associated with SBO [23,24,100,101] (Figure 11). Small bowel adenocarcinoma can appear on CT as short focal or segmental irregular and circumferential wall thickening, resembling apple core lesions [23,24,29,30]. Sustained neoplastic malignant intestinal stenosis and adenocarcinoma may be associated with chronic signs of SBO, such as a high-grade obstruction with stasis [90,100,101].

Carcinoid intestinal tumors can also present with SBO due to mesenteric fibrosis, which is often associated with local invasion [25,26,27,91,92,93]. On CT, it is usually possible to identify mesenteric and vascular impairment, as well as closed-loop or strangulated obstructions in the context of SBO or vascular impairment associated with neoplastic infiltration [91,92,93].

**Figure 12:**
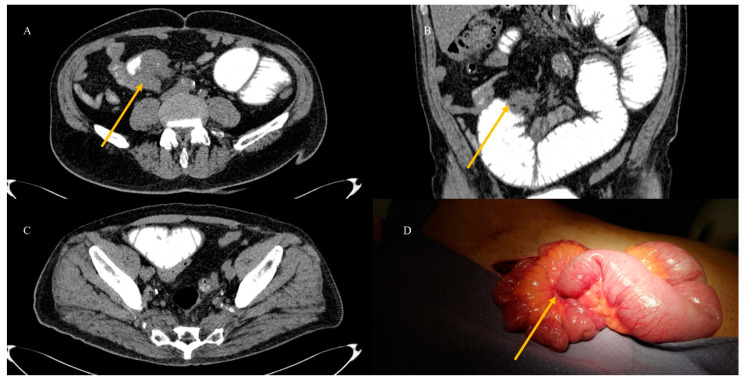
This figure shows a CT scan with GGF, which confirms the irregular stenotic thickness of a distal jejunal loop (orange arrow) in image (**A**). In image (**B**), irregular tissue in the mesenteric fat (orange arrow) is visible, along with a high grade of obstruction seen in both images (**B**,**C**) where GGF transit is stopped. Prompt surgical intervention confirmed the neoplastic stenosis (orange arrow) in image (**D**), resulting in an invasive intestinal adenocarcinoma.

A CT with GGF may be useful in identifying neoplastic stenosis in cases where endovenous contrast administration cannot be performed or as an integration of a previous CECT (Figure 12). This can help in the visualization of the small bowel lumen, stenotic segments, and oral contrast progression [40,41].

**Figure 13:**
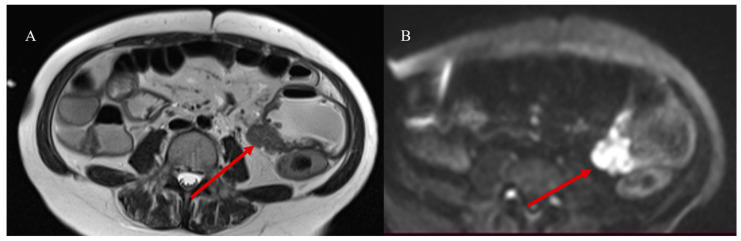
MRI of a 28-year-old woman with persistent abdominal pain and sub-occlusive crisis revealed concerning findings. In image (**A**), the T2 HASTE sequence highlights the presence of mild hyperintense irregular tissue (red arrow) with proximal large jejunal dilatation, indicating persistent stenosis. In image (**B**), the DWI sequence shows marked restriction in the irregular thickness, suggesting a malignant nature. Histology confirmed the presence of intestinal lymphoma.

MRI offers the advantage of tissue characterization through multiparametric study [54,55,56,57,58,59] (Figure 13). The coronal cine-balanced SSFP can be useful in assessing small bowel mobility, low-grade stenosis, and the level of obstruction [54].

### 2.5. Small Bowel Tumors Presenting with Bleeding, Ischemia, and Perforation

**Figure 14:**
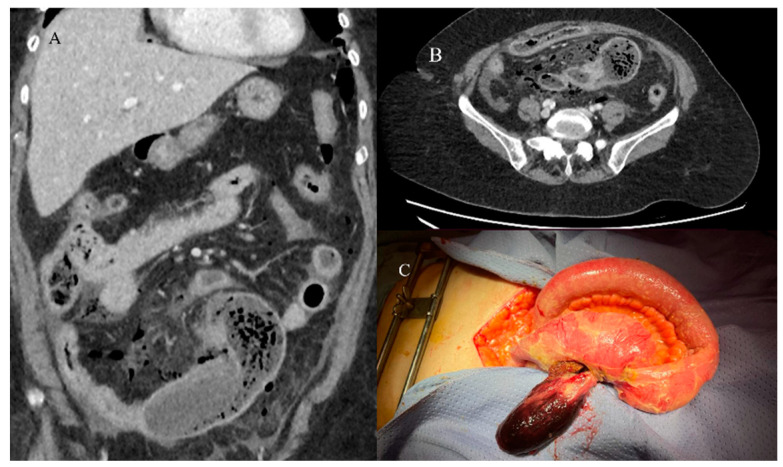
CT images from the previous case of the 72-year-old woman with an intestinal polyp and SBO show signs of necrosis and perforation. Initially, nonsurgical management was decided upon; however, the intestinal polyp became complicated with necrosis and perforation. Signs of perforation with free air are visible on the coronal plane in image (**A**) and on the axial plane in image (**B**). During surgery in image (**C**), a necrotic ileal polyp with intestinal laceration was found.

Different studies have described SBTs presenting in emergencies with major vascular complications, such as bleeding, ischemia, and perforations [14,17,18,19]. GI bleeding is reported as the most common complication of GISTs as they can frequently be associated with mucosal ulcerations [33]. NETs can also manifest as GI bleeding [26]. Perforations may be caused by tumor vascular embolization, increased intraluminal pressure due to obstruction, tumor necrosis caused by tumor cell replacement, or chemotherapy [102]. The most common malignancies found after the histopathologic examination of perforated small bowel specimens include lymphoma, leiomyosarcoma, GISTs, adenocarcinoma, and metastatic carcinomas of the hypopharynx, cervix, and lung [24,103] (Figure 8). In some rare cases, inflammatory polyps associated with small bowel ischemia and perforation have been reported [104] (Figure 14).

The desmoplastic reaction usually associated with carcinoid tumors, caused by the effect of serotonin or other vasoactive hormones produced by the tumor, may damage the mesenteric vessels, causing small bowel ischemia [26]. CECT is usually the best tool to use in emergencies to rule out the vascular complications (ischemia and bleeding) of SBTs, as well as perforations in hemodynamically stable patients [104,105].

Extravasation of iodine contrast material on CECT is usually indicative of active arterial bleeding. On the other hand, CTE can improve the detection of the location of GI bleeding and has high sensitivity in detecting SBTs presented with obscure GI bleeding [26,33,39,104]. With the use of DECT, it is possible to use a smaller volume of contrast, and the utilization of virtual unenhanced imaging can also eliminate the need for multiphase scanning [42,44,45].

CECT is the modality of choice for localizing the site of perforation, with an accuracy ranging from 82% to 90% [105]. GGF may be useful in emergencies in the presence of SBTs with perforations as contrast leakage is a highly specific sign for localizing the site of perforation; however, it has a low sensitivity, ranging from 19% to 42% [105]. It should never be used in cases of suspected GI bleeding as it can be masked by the intraluminal contrast of GGF. However, in patients with obscure GI bleeding, MRE has high accuracy in characterizing the underlying diseases as inflammatory or tumoral conditions [52].

### 2.6. Small Bowel Tumors Mimicking Inflammatory Disease

**Figure 15:**
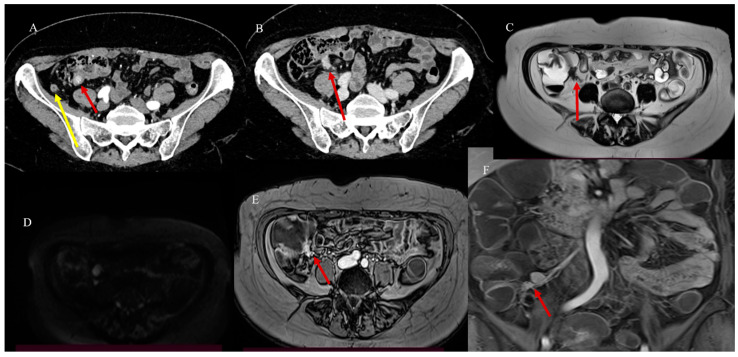
In images (**A**,**B**), the abdominal CECT of a 54-year-old patient with a previous history of breast cancer and acute abdominal pain on the right flank is shown. In image (**A**), there is a mild thickening of the appendix (yellow arrow). Additionally, an earlier arterial enhancement was noted in the distal ileum at the level of the ileocecal valve (short red arrow). An irregular nodule with an early enhancement was also observed (long red arrow) in the near adipose tissue. The MRE was performed to integrate and confirm hypointense irregular tissue near the ileocecal region on the T2 HASTE sequence (short red arrow), as seen in image (**C**), with some lymphadenopathy showing restricted diffusion in image (**D**). An earlier enhancement of the irregular tissue is visible (short red arrow) in images (**E**) in T1 vibe Dixon sequence after contrast administration on the axial plane. Small, irregular tissue (short red arrow) with lymphadenopathy is also visible in image (**F**). A small NET was suspected on imaging and confirmed using histology.

Diagnosing SBTs in emergency situations can be challenging as they may mimic inflammatory diseases or even coexist with them [24,29,106] (Figure 15).

Patients with CHD and CD are particularly at risk of developing small bowel cancers [6,7,8,9,10]. The disruption of the intestinal barrier’s homeostasis can lead to pathological interactions among epithelial cells, microflora, and the immune system [11,12].

Patients with CHD face a higher risk of developing small bowel adenocarcinoma, especially in the ileum [23,29]. The ileocecal region is a common site for primary intestinal lymphoma, which can also mimic IBD [31,107].

**Figure 16:**
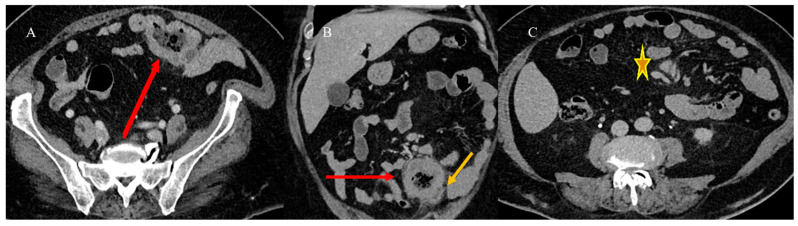
Abdominal CECT scan of a 70-year-old patient with a history of LS who presented to the emergency room with abdominal pain and a mild fever. In image (**A**), there is a visible dilatation of a jejunal loop with severe parietal thickness (red arrow) and fecal stasis, and it appears to be simulating an abscess. However, this irregular thickness and dilatation can also be seen in malignant tumors such as lymphoma. In image (**B**), there is an irregular dilation of the jejunal loop (short red arrow) with an adjacent small collection (orange arrow). Additionally, some lymphadenopathy is visible in image (**C**) (orange star with yellow outline). It is challenging to differentiate between lymphoma and adenocarcinoma tumors with an inflammatory collection based solely on imaging.

**Figure 17:**
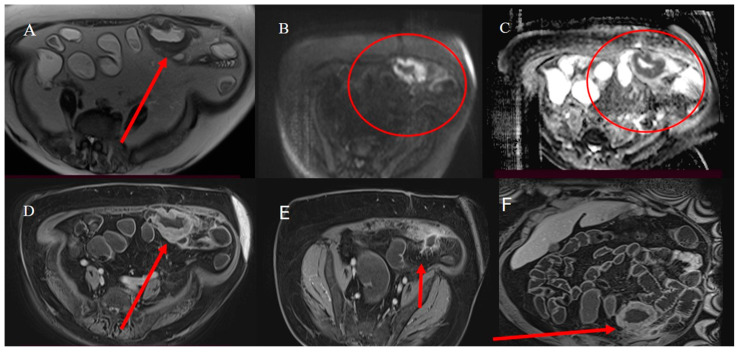
MRE integration of the previous case confirmed, in the T2 HASTE sequence, a hypointense parietal thickness with dilation of the jejunal loop (red long arrow) in image (**A**). This dilation showed a marked reduction in restriction, as seen in image (**B**) (red circle), with a lower ADC value in image (**C**) (red circle). Homogeneous enhancement is also visible in the T1 vibe Dixon sequence after contrast administration in image (**D**) (red long arrow). Additionally, a small posterior collection is visible (short red arrow) in image (**E**), along with focal dilatation on the coronal plane in image (**F**) (red arrow). The histology found an invasive adenocarcinoma.

Small bowel adenocarcinoma, lymphoma, and malignant GISTs can present with similar radiological features, appearing as large soft-tissue masses connected to the bowel lumen or as a pseudoaneurysmal small bowel dilation [23,24,31,33] (Figure 15). This presentation can resemble an abscess or an inflammatory disease on CECT [23,24,29,107]. However, MRI can help in characterizing the underlying disease (Figure 16). Small bowel lymphomas typically exhibit higher DWI values and a lower signal on ADC maps [21,24,57,58,59]. However, in some cases, it is very difficult to make a diagnosis based solely on imaging (Figure 17).

### 2.7. Metastatic Presentation of Small Bowel Tumors

**Figure 18:**
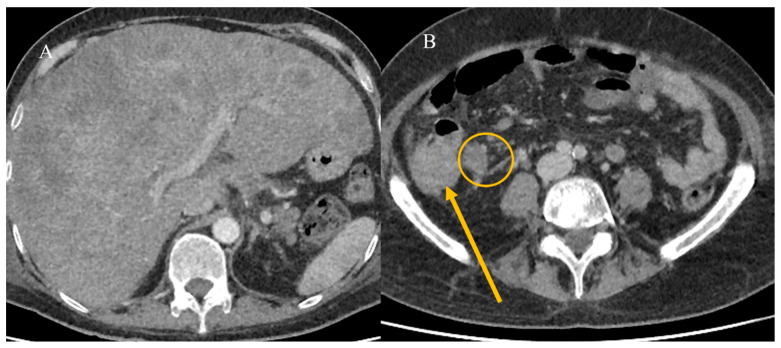
During a CECT scan conducted in an emergency situation, multiple liver metastases were discovered. These metastases are clearly visible in image (**A**), showing a significant thickness near the ileal–cecal region (indicated by the orange arrow) in image (**B**). Additionally, multiple infiltrating tissue nodules were identified in the perivisceral fat (indicated by the orange circle). A biopsy of the liver metastases confirmed that they originated from an intestinal carcinoid tumor.

Malignant SBTs can present in emergency situations with advanced and metastatic disease. In a study by Farhat et al. [18], almost half of the patients presented with metastatic disease (stage III/IV). Lymph nodes were the most common site involved in metastasis, followed by the liver, lung, brain, bone, and omentum. Approximately one-third of patients with small bowel adenocarcinoma can present with metastatic disease, and the common sites of metastasis are the liver and peritoneum [30]. Small bowel carcinoid tumors can have a metastatic presentation in 21% of cases, and it is usually associated with the histologic grade of tumors that show deep invasion through the muscolaris propria [26]. Liver metastases are reported in gastrointestinal NETs in 37–55% of cases [26] (Figure 18).

**Figure 19:**
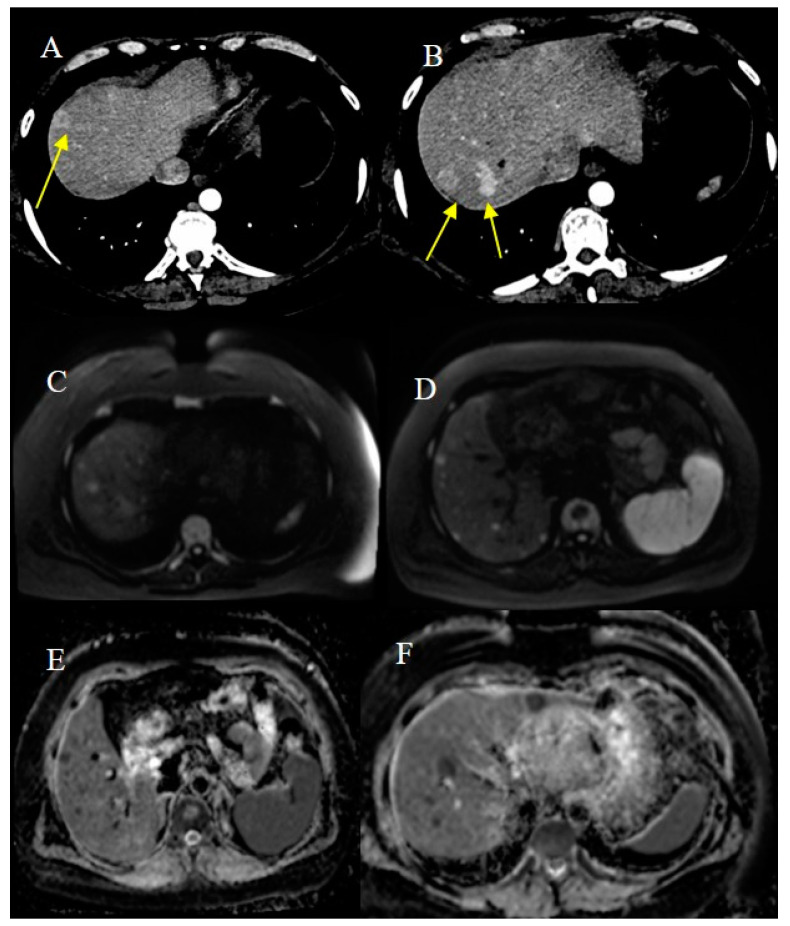
A case of hypervascular liver metastasis (yellow arrow) on contrast-enhanced CT (CECT) from an ileal neuroendocrine tumor (NET) is shown in images (**A**,**B**). In the MRI diffusion-weighted imaging (DWI) sequence with a higher b value, more liver metastases are visible as hyperintensity signals in images (**C**,**D**), with lower apparent diffusion coefficient (ADC) values in images (**E**,**F**).

Therefore, a dedicated triple-phase contrast study with a good arterial phase on CECT or gadolinium dynamic MRI is important in the evaluation of liver metastases as they can only be seen in the arterial phase [26]. MRI is reported to have a greater sensitivity than CT for the detection of liver metastases, especially for smaller lesions and in the presence of liver steatosis [108,109]. In particular, DWI sequences increase the detection of small liver metastases (Figure 19) and can be very useful in patients with compromised renal function who cannot receive intravenous gadolinium-based contrast agents [108]. However, the sensitivity of MRI increases when DWI is combined with multiphase contrast-enhanced MRI protocols. The use of hepatocyte-specific contrast agents usually provides a better contrast-to-noise ratio and improves interobserver reliability for the measurement of NET liver metastases compared with extracellular contrast agents [63].

**Figure 20:**
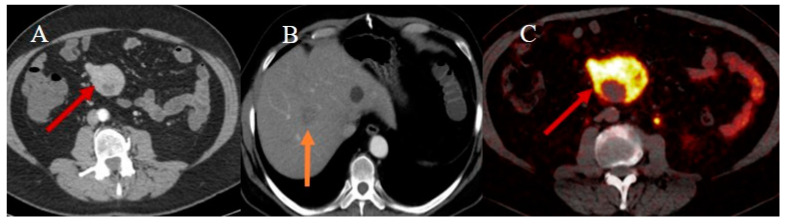
Image (**A**) displays a contrast-enhanced computed tomography (CECT) image of a 75-year-old patient who came to the emergency room with abdominal pain. A hypervascular ileal mass was discovered (red arrow) during the examination. The patient also presented with liver metastases (orange arrow), one of which is depicted in image (**B**) (orange arrow). The ileal mass exhibited increased uptake of 68Ga-DOTA PET/CT as visualized in image (**C**) (red arrow) and was determined to be compatible with an ileal neuroendocrine tumor (NET).

FDG PET/CT offers an advantage over other modalities in metastatic staging, assessing necrosis, and detecting liver metastases. 68Ga-DOTA PET/CT for small bowel NETs can be useful for initial tumor diagnosis, staging, and the detection of liver metastases [62,63,64,110] (Figure 20).

## 3. Conclusions

In conclusion, we summarized the versatile presentations of SBTs, particularly in emergency situations, where CT imaging plays a crucial diagnostic role in ruling out complications such as SBO, perforations, and metastatic presentations. MRI and MRE can also play an important role in tissue characterization in non-emergency cases, providing a non-invasive way to characterize SBTs. FDG PET/CT is useful for staging metastatic disease, while 68Ga-DOTA PET/CT is beneficial for detecting and staging small bowel NETs.

Looking ahead, with the increasing use of artificial intelligence and radiomics, it will become possible to accurately detect small SBTs and analyze the morphological and quantitative features of each imaging modality. By integrating biological data with morphological imaging data, we can identify biomarkers that indicate prognosis and disease progression, ultimately improving patient management.

## Figures and Tables

**Table 1:** This table summarizes the main clinical indications for each imaging modality in the evaluation of SBTs, along with the protocols used, advantages, and limitations of each technique (CECT: contrast-enhanced computed tomography; CTE: CT enterography; DECT: dual-energy CT; IUS: intestinal ultrasound; GGF: Gastrografin; MDCT: multidetector CT; MRE: magnetic resonance enterography; PET: positron emission tomography).

	Indications	Protocols	Advantages	Limitations
Abdominal X-Ray	In the emergency department for acute abdomen with SBO or small bowel perforations	Anterior–posterior (AP) supine projection or PA prone, lateral decubitus, upright AP, and lateral cross-table (with the patient supine)	Low dose; easier availability; cost-effective	Poor sensitivity for SBO causes and for SB parietal evaluation
IUS	In pediatric patients; in patients with GI diseases, including IBD and GI obstruction	The small bowel is explored with a medium- or high-frequency convex or lineal transducer (5–15 MHz)	Radiation-free; non-invasive; cost-effective	The experience of the operator, artifacts produced by intestinal gas, and the patient’s body habitus, including BMI and the thickness of the fatty layer of the anterior abdominal wall
CT				
CECT	In an emergency and initial staging of SBTs	A good arterial phase (30–35 s) for higher-vascular SBT tumors as NETs and GI bleeding	Speed; easier availability; MPR reconstruction	Small intestinal tumors may be not visualized without distension; allergy to contrast agents
CT with GGF	In an emergency; in cases of SBO that do not require emergency surgery; for transit evaluation in SBTs with SBO; in cases of small bowel perforations	100 mL of GGF diluted into 50 mL water via NG tube or taken orally	GGF can aid in the management of SBO, has a therapeutic effect in the conservative management of adhesive SBO, and can depict the presence of intestinal fistulas	Can mask GI bleeding in the intestinal lumen
CTE	To diagnose and stage SBTs; integration with previous CECT in non-emergency cases	Small bowel distension through negative or positive oral contrast agents; split bolus technique: contrast agent injected twice, with the first injection consisting of 60% of the total dose, followed by injection of the remaining 40%	Improved visualization of the small bowel wall and lumen; enhanced detection of small intestine lesions and multiple localizations	Inadequate small bowel distention; less sensitive than capsule study to detect small bowel distension; cannot be tolerated by some patients
DECT	To diagnose and stage SBTs; in emergencies when available	Use of two different X-ray tube potentials to acquire images simultaneously or sequentially	A combination of low-energy monochromatic images, iodine maps, and virtual unenhanced images improves lesion detection and characterization; reduction of radiation dose and number of CT scans	Higher costs; lesser availability than conventional MDCT
MRI				
MRE	For SBT tissue characterization in non-emergency cases or in stable patients	Intestinal distension with oral biphasic contrast agents, with an optimal volume of 1000–1500 mL that can be ingested over 45–60 min before the examination; T2 HASTE with and without FS on axial and coronal plane; Trufi T2 on axial and coronal plane; DWI; VIBE T1 FS pre-contrast on coronal plane; if stenosis is present, it may be helpful to use the cine-balanced sequence	Tissue characterization through multiparametric sequence; DWI can be used to predict and monitor SBTs; DWI is more sensitive to detecting LI metastasis in liver steatosis	Claustrophobia; PMK not MRI-compatible
PET/CT	Staging and restaging of SBTs	2-deoxy-2-^18^F-D-glucose (FDG) is the most commonly used tracer; 68-Gallium (68Ga)-DOTA peptide (DOTATATE, DOTATOC, and DOTANOC) PET/CT is used in small bowel NETs; full body scan acquisition	Ability to detect local and distant metastatic disease; 68-Gallium (68Ga)-DOTA peptide has higher sensitivity and specificity for small bowel; NETs tumor localization, staging, and receptor status assessment; 68-Gallium DOTATATE PET/CT is accurate for detecting initial or recurrent NETs in patients with carcinoid-like symptoms and negative anatomical imaging	PET/CT can produce false positives in patients with inflammation or infection; PET/CT has less spatial resolution than CT and MRI; PET and CT images can be misregistered due to the small bowel’s mobility

## Data Availability

Data sharing is not applicable.
